# Improving Biosurveillance Systems to Enable Situational Awareness During Public Health Emergencies

**DOI:** 10.1089/hs.2016.0097

**Published:** 2017-02-01

**Authors:** Jennifer B. Nuzzo

Over the past 10 years, US health security has been threatened by a series of infectious disease events: the 2009 influenza outbreak, the emergence of Middle East respiratory syndrome (MERS), the Ebola outbreak in West Africa, and the rapid spread of Zika virus throughout the Americas. In each of these events, inadequate information has delayed initial detection of the outbreak, and a lack of understanding about the underlying epidemiology of the viruses hindered control efforts. As the ongoing US response to Zika illustrates, delays in detecting and responding to outbreaks can exacerbate their human and economic tolls. While the United States continues to struggle to understand how Zika virus can spread and cause serious disease, more than 37,000 cases have already been identified in the United States and its territories.^1^ Each one of these cases must be investigated by health authorities to ensure that they do not spread their infection. In particular, pregnant women must be followed closely, as Zika can cause fetal death or severe birth defects. It is estimated that each baby who is born with Zika-related birth defects will cost families and the US healthcare system up to $10 million.^2^

The continued challenge that the United States faces in detecting and knowing how best to respond to acute infectious disease threats like Zika should encourage a closer analysis of our current biosurveillance capabilities and spur action toward much-needed, sustainable improvements. Biosurveillance generally refers to the continued monitoring of information sources for the purposes of detecting and managing an outbreak or other public health event, whether naturally occurring or deliberate. Biosurveillance systems may gather and analyze data from a variety of human, animal, plant, and environmental health sources. The goal of this activity is to provide situational awareness—an understanding of what is going on—with respect to the occurrence of biological threats and to guide efforts to control them.

Since 2001, the federal government has made considerable investment in deploying biosurveillance systems across the country with the goal of providing early detection of and situational awareness during public health emergencies. Federal support has vastly improved the state of surveillance in the United States. The nation now benefits from having a network of more than 150 public health laboratories that can detect a biological attack and outbreaks of other diseases of public health significance.^3^ Additionally, the majority of health departments in the United States now have a mix of traditional disease-specific and more timely syndromic surveillance systems in place to monitor disease trends and to provide situational awareness during public health emergencies.

Despite these important advances, there remain fundamental gaps in the ability of existing biosurveillance systems to support real-time decision making during public health emergencies. In 2012, the White House attempted to address these shortcomings by releasing the National Biosurveillance Strategy. This document established the important expectation that biosurveillance systems will be able to (1) rapidly alert and inform decision makers of potential incidents of national significance; (2) continually provide critical updates as circumstances evolve; and (3) access information that answers decision makers' questions about probable impacts of an event and the consequences of action/inaction.

The National Biosurveillance Strategy did not offer details on how this vision for biosurveillance was to be implemented, but it called for the development within 120 days of an Implementation Plan. To date no such plan has been released.

As a matter of priority, the next Administration should work to improve our national biosurveillance capabilities in the following ways.

## Recommendations

➢ **Enhance support for state and local health departments.**

State and local surveillance programs are the foundation of our national biosurveillance enterprise. The federal government's capacity to detect and maintain situational awareness during catastrophic events depends on the ability of state and local agencies (particularly health departments) to build and maintain robust and flexible biosurveillance systems. However, national biosurveillance capabilities are threatened by shortfalls in state and local financial resources. Limits in state budgets make it difficult for agencies to maintain information systems and staff solely with local resources. The Centers for Disease Control and Prevention (CDC) public health emergency preparedness cooperative agreements have been essential sources of support for state and local health departments to enable them to develop and maintain basic biosurveillance capabilities. While federal preparedness investments have been an important first step, the level of federal funding for biosurveillance appropriated to date is not commensurate with the strategic national importance of these systems. Failure to increase support for health departments will erode critical progress made to date toward ensuring that basic biosurveillance capabilities exist across the United States.

Above all, the US government must ensure that there are adequate numbers of competent personnel to run biosurveillance programs, particularly at state and local health departments. Unfortunately, continued cuts in federal preparedness funding over the past 10 years have forced state and local health departments to scale back on important preparedness programs. These cuts, combined with state budget deficits and layoffs, have exacerbated existing shortages of highly skilled and competent public health personnel to build and maintain biosurveillance systems. Ensuring the functioning of critical biosurveillance programs will require sustained and committed federal support for biosurveillance programs, including adequate and flexible funding to hire and retain biosurveillance analysts.

➢ **Provide critical electronic health record data to public health departments.**

Real-time exchange of patient-level data between clinical and public health communities is critical for detecting and responding to public health emergencies. Public health departments need detailed data about patients who are affected in order to understand key aspects of the outbreak. While there has been a concerted effort and significant funding allotted to developing electronic health records and to encouraging their adoption by medical providers, far less attention and funding support have been allocated to making sure the information contained in electronic health records is available to public health departments. While some public health departments may receive some electronic data from electronic health records, such as immunization records and laboratory reports, few have the ability to access electronic health records in real time and in a flexible, query-based way that is likely to provide the answers necessary to quickly get on top of a public health emergency as it unfolds.

As an immediate priority, the HHS Office of the National Coordinator for Health Information Technology (ONC) should work with CDC and the Centers for Medicare and Medicaid Services (CMS), together with input from state and local public health stakeholders, to define and implement national data standards to improve public health departments' access to electronic health records during public health emergencies. CDC should work with state and local stakeholders to determine how to best access and use essential public health data contained in electronic health records in a manner that assures the security and confidentiality of patient information.

➢ **Assess the ability of biosurveillance systems to support decision making.**

The next Administration should preserve the National Biosurveillance Strategy and release from the White House a plan for its implementation. At a minimum, the implementation plan should contain an analysis of where existing biosurveillance systems fail to provide sufficient information to support decision making. To complete this analysis, US agencies should work with states to define a minimum set of information that will be needed to manage a public health emergency and then map this information against existing biosurveillance systems.

This analysis will likely identify deficits in the ability of existing surveillance systems to provide logistical information needed for mounting a response to a public health emergency. Experience in previous emergencies has shown that while most public health surveillance systems focus on data related to the number of infected patients, they often lack data pertaining to availability of hospital beds, pharmaceuticals, personal protective equipment, and other medical supplies that are necessary to make informed decisions about how best to respond to a public health emergency. The US government could bring these and other information needs to the private sector and identify potential ways to collect that data and create data-sharing provisions (eg, confidentiality agreements, de-identification steps, etc) needed to share this information with response agencies.

➢ **Integrate animal, environmental, and human health data.**

The recent Ebola epidemic in West Africa demonstrated an important deficiency in biosurveillance: the integration of human, environmental, and animal health surveillance data. Though the outbreak caught many political leaders and public health experts by surprise, existing wildlife data indicated that a human outbreak in the region was possible.^4^ Despite known important linkages between animal and environmental health in determining the occurrence of events, like the Ebola epidemic, that threaten human health, there continue to be insufficient efforts to integrate knowledge and data from across these sectors.

The US government should work to improve the integration of human, animal, and environmental data. An important first step will be to increase support for existing surveillance programs that have already demonstrated success, such as ArboNET, which provides important data on the occurrence of Zika, West Nile, and other viruses. Additionally, the US government should give serious consideration to the recommendation, which was made by the bipartisan Blue Ribbon Study Panel on Biodefense, to create a Nationally Notifiable Animal Disease System.^5^ Finally, the US government should improve the integration of the food-related surveillance initiatives that exist across the federal government. There are many different, separate national surveillance systems that, if integrated, could provide a better understanding of the occurrence and possible causes of foodborne illness outbreaks. Federal agencies should digitally connect and automate the comparisons of data from the food, animal, and human health surveillance programs that are operated by CDC, FDA, and USDA, which may provide an earlier indication of a link between human and animal infections. At the very least, there should be a way to directly compare isolated patterns that are in animal and human health surveillance programs. CDC's PulseNet and USDA's VetNet programs should be linked and equipped to automate analysis of these 2 data streams for evidence of similarities that may indicate a common exposure.

➢ **Enhance laboratory capacity and improve the availability of diagnostics tools.**

As we have seen in the current Zika crisis, the ability of the United States to conduct surveillance for and respond to emerging infectious disease outbreaks is severely compromised when there are insufficient tools for accurately determining who has been infected. It has been reported that because of laboratory backlogs, pregnant women in Florida have experienced delays in getting the results from tests to determine if they have been infected by Zika. Diagnostic delays such as these not only compromise care for women and their unborn children, but hinder our abilities to conduct epidemiologic investigations to determine whether and how the outbreak may be spreading. Increased support for public health and clinical laboratories is needed to ensure there is adequate surge capacity during crises.

The current Zika crisis also underscores that new diagnostic technologies are needed. Currently, it is not easy to determine if someone has been infected with Zika unless they are within a small window of time after the onset of symptoms. The inability to determine with confidence whether someone was infected with Zika in the past makes it difficult to counsel patients and interrupt transmission. The US government should expand current support for efforts to develop new diagnostic technologies for Zika and other important public health threats.

➢ **Promote open data.**

During one of Europe's deadliest *E. coli* outbreaks, sequence data from the outbreak strain was shared publicly, and crowd sourcing enabled the data to be analyzed more quickly than traditional approaches would have allowed. While the world is undergoing a big data revolution, in the United States a current bottleneck in biosurveillance analysis is lack of good tools and a skilled workforce to support better analysis and visualization of biosurveillance information, particularly data that exist outside of traditional public health surveillance streams. The United States should take advantage of the wisdom and talent of the crowds to improve the analysis and visualization of biosurveillance information by making anonymized surveillance data publicly available. Publishing de-identified surveillance data in as close to real time as possible would enable interested citizens to develop applications to enable enhanced analysis of biosurveillance information.

## Conclusion

What the past decade has shown us is that the continued occurrence of infectious disease events is all but inevitable. As the pace of globalization and international travel increases, new diseases will continue to emerge and spread. Our best defense against these events is to have in place systems that will quickly detect their occurrence and to improve understanding of what is needed to stop them. Without effective biosurveillance systems, political leaders will have little information to guide decisions about what measures should be taken to ensure that small outbreaks don't go on to become costly epidemics and what measures would be likely to exacerbate the toll of the event. Though important strides have been made toward development of these systems in the United States, critical gaps remain, and eroding financial support for biosurveillance threatens to undermine progress made to date. To ensure it has the best information available to successfully manage the infectious disease events that will inevitably occur during the next administration, the US government should expand its support for the biosurveillance mission.

## References

[1] Zika virus: case counts in the US. Centers for Disease Control and Prevention website. Updated 11 23, 2016 http://www.cdc.gov/zika/geo/united-states.html Accessed 1130, 2016

[2] Frieden T, Fauci AS CDC and NIH officials: how not to fight the Zika virus. Washington Post 8 31, 2016 https://www.washingtonpost.com/opinions/cdc-and-niaid-officials-congress-is-showing-how-not-to-fight-the-zika-virus/2016/08/31/c2efc146-6f7f-11e6-8365-b19e428a975e_story.html Accessed 1130, 2016

[3] Khan AS Public health preparedness and response in the USA since 9/11: a national health security imperative. Lancet 2011;378(9794):953-956 21890060 10.1016/S0140-6736(11)61263-4 PMC7135567

[4] Hayman DT, Yu M, Crameri G, et al. Ebola virus antibodies in fruit bats, Ghana, West Africa. Emerg Infect Dis 2012;18(7):1207-1209 22710257 10.3201/eid1807.111654 PMC3376795

